# Interplay between *Plasmodium falciparum* haemozoin and l-arginine: implication for nitric oxide production

**DOI:** 10.1186/s12936-018-2602-0

**Published:** 2018-12-06

**Authors:** Yolanda Corbett, Sarah D’Alessandro, Silvia Parapini, Diletta Scaccabarozzi, Parisa Kalantari, Stefania Zava, Flavio Giavarini, Donatella Caruso, Irma Colombo, Timothy J. Egan, Nicoletta Basilico

**Affiliations:** 1 Dipartimento di Scienze Farmacologiche e Biomolecolari, Università degli Studi di Milano, 20133 Milan, MI Italy; 2 Dipartimento di Bioscienze, Università degli Studi di Milano, 20133 Milan, MI Italy; 3 Dipartimento di Scienze Biomediche, Chirurgiche e Odontoiatriche, Università degli Studi di Milano, 20133 Milan, MI Italy; 4 Department of Immunology, Tufts University School of Medicine, Boston, MA 02111 USA; 5 Department of Chemistry, University of Cape Town, Private Bag X3, Rondebosch, 7701 South Africa

**Keywords:** Malaria, *Plasmodium falciparum* haemozoin, Macrophages, l-Arginine, Inducible nitric oxide synthase, Nitric oxide

## Abstract

**Background:**

*Plasmodium falciparum* haemozoin, a detoxification product of digested haemoglobin from infected erythrocytes, is released into the bloodstream upon schizont rupture and accumulates in leukocytes. High levels of haemozoin correlate with disease severity. Some studies have shown that concentrations of the substrate of inducible nitric oxide synthase (iNOS), l-arginine, as well as nitric oxide are low in patients infected with *P. falciparum* malaria. The present study investigates, in vitro, the role of *P. falciparum* haemozoin on nitric oxide production, iNOS expression in macrophages, and the possible interaction between l-arginine and haemozoin.

**Methods:**

*Plasmodium falciparum* haemozoin was obtained from in vitro cultures through magnetic isolation. Phagocytosis of haemozoin by immortalized bone marrow derived macrophages was detected by confocal reflection combined with fluorescence microscopy. Nitrite concentrations in the supernatants was evaluated by Griess assay as a standard indication of nitric oxide production, while iNOS expression was detected on cell extracts by western blotting. Detection of l-arginine in haemozoin-treated or untreated media was achieved by liquid chromatography–tandem mass spectrometry (LC–MS/MS).

**Results:**

Haemozoin synergizes in vitro with interferon-gamma to produce nitric oxide. However, when mouse macrophages were stimulated with haemozoin, a proportional increase of nitric oxide was observed up to 25 μM of haemozoin, followed by a decrease with doses up to 100 μM, when nitric oxide release was completely abrogated. This was not due to reactive oxygen species production, nor to an effect on iNOS activity. Interestingly, when at 24 h, haemozoin-treated macrophages were washed and incubated in fresh medium for further 24 h, the nitric oxide production was restored in a dose–response manner. Similar results were seen when l-arginine-enriched media was used in the stimulation. Moreover, muramyldipeptide, a strong nitric oxide inducer, was unable to activate macrophages to release nitric oxide in the presence of haemozoin-treated medium. By LC–MS/MS a complete depletion of l-arginine was observed in this haemozoin-treated, conditioned medium.

**Conclusions:**

It is proposed that haemozoin interacts with l-arginine reducing its availability for iNOS, and thus decreasing nitric oxide production. The clinical (or pathological) implications of these results are discussed.

**Electronic supplementary material:**

The online version of this article (10.1186/s12936-018-2602-0) contains supplementary material, which is available to authorized users.

## Background

Malaria remains a major threatening problem worldwide with an annual burden of 216 million cases in 2016 and 445,000 deaths [1], especially young children, in Africa, and pregnant women. The severe form of the disease is characterized by adherence and sequestration of infected erythrocytes to the vascular endothelium, associated with excessive inflammatory response [2]. Nitric oxide (NO), an innate immune mediator commonly released during malaria infection, seems to modulate the sequestration of parasitized erythrocytes [3–5]. Indeed, protection from severe malaria has been shown in individuals with specific genetic disorders (i.e. mutations) that promote NO production [6]. In severe malaria cases, low levels of NO are present, as well as low levels of its precursor, l-arginine (l-Arg) [7–9], which is also associated with a reduced erythrocyte deformability [10]. It is also reported that the management of patient by treatment with l-Arg [7] or by inhaled NO as adjunctive therapy improves NO bioavailability, leading to reduction of the symptoms of severe malaria [11, 12]. Similar observation was made in murine experimental models of cerebral malaria [13–16]. However, contrasting data were also published. A pilot study presented by Yeo and colleagues showed that l-Arg treatment in a small cohort of malaria patients did not induce changes in endothelial NO bioavailability or lactate clearance, a marker of disease [17]. Also, inhaled NO therapy failed to improve the levels of angiopoietin-1, a biomarker of malaria severity [18].

Coincidentally, there is an association between severe malaria and *Plasmodium falciparum* haemozoin (HZ), a by-product of digested haemoglobin from *P. falciparum* infected erythrocytes, released into the bloodstream upon cell rupture [19–22]. Increase of l-Arg breakdown (free or intra-erythrocyte) has been reported elsewhere [23], while another report shows that l-Arg inhibits the formation of the synthetic pigment crystal (beta-hematin, βH) [24]. There is no information so far about a possible interaction between l-Arg and HZ. The aim of the present study was to investigate in vitro the role of HZ in the modulation of NO or iNOS, and the role of l-Arg in this phenomenon.

## Methods

### Reagents

CellMask™ Plasma Membrane Stain and DAPI were obtained from Molecular Probes/Invitrogen (Thermo Fisher Scientific, Waltham, MA, USA). Protein Extraction Buffer (RIPA Buffer) and protease inhibitor cocktail were obtained from Axxora (USA). Polyclonal anti-iNOS antibody was obtained from Cell Signaling (Danvers, MA, USA). Mouse monoclonal anti-actin antibody, horseradish peroxidase (HRP)-conjugated donkey anti-rabbit, and goat anti-mouse antibodies were obtained from Santa Cruz Biotechnology Inc. (Dallas, TX, USA). Filtration supplies were from Millipore^®^ (Milan, Italy). Unless otherwise stated, chemical reagents were obtained from Sigma-Aldrich (Milan, Italy), while cell culture media, serum and supplements, from EuroClone (Milan, Italy).

### Culture of bone marrow derived macrophages

Immortalized mouse C57Bl/6 bone marrow derived macrophages (BMDM) from wild type lineage were generated and maintained in the growth medium, as previously reported [25, 26]. The basic composition of the growth medium used for BMDM consisted in Dulbecco’s minimal essential medium, DMEM, which contains 0.084 g/l of l-Arg, supplemented with l-glutamine (2 mM), HEPES (20 mM, pH 7.3), and 10% heat-inactivated fetal bovine serum (FBS) (South America origin EU Approved).

### Culture of *P. falciparum* and preparation of haemozoin

*Mycoplasma*-free *P. falciparum* parasites (strain 3D7) were maintained in vitro as previously described [27, 28]. For malaria cultures, fresh erythrocytes where obtained from AVIS Comunale Milano with the consent of healthy donors. The parasites were kept in culture at 5% haematocrit (human type 0 + erythrocytes) at 37 °C in RPMI 1640 medium supplemented with 10% heat-inactivated 0 + human plasma, 20 mM HEPES, pH 7.4, in a standard gas mixture consisting of 1% O_2_, 5% CO_2_, 94% N_2_.

The methodology to isolate HZ has been standardized, applied and characterized in our previous report [28], with some modifications. Briefly, HZ was isolated using a LS column (MACS, Miltenyi^®^) from *P. falciparum* cultures the day after parasitaemia reached > 5% trophozoites. Cultures at low hematocrit (~ 0.5%) were placed onto the column, and the free-HZ was isolated. By using the MACS-LS and low haematocrit, only free HZ was collected and not the parasitized erythrocytes. HZ was pelleted, resuspended in PBS, finely dispersed with a 1-ml syringe, and stored at 4 °C. To remove free haem and contaminating erythrocytes, HZ was extensively washed with PBS and hypotonic solution. The absence of free haem was confirmed by reading the OD at 405 nm (Soret band) in the supernatants obtained after HZ washing. Also, all HZ preparations were endotoxin-free as checked by the *Limulus* amoebocyte lysate-based assay, as previously described [28].

Stocks of HZ were made by pooling different preparations. Being HZ a crystal of haem molecules bound together, its molar concentration was calculated as haem equivalent, referring to the molecular weight of haem, as previously reported [28]. Briefly, aliquots from HZ pools were diluted in 1 M NaOH, in order to obtain the heme monomers in solution. A standard curve of hemin was also prepared in NaOH 1 M. The samples were read spectrophotometrically at OD 405 nm (Synergy 4, Biotek^®^ microplate reader) and the concentration of haem monomers in the HZ samples was extrapolated from the standard curve. The doses of HZ used for in vitro stimulations were chosen as relevant to biological conditions, and calculated considering a parasitaemia of 1–4% and the iron content of trophozoites, as reported earlier [28–30].

### Confocal microscopy

BMDMs were incubated with HZ (100 µM) in a 35-mm glass bottom tissue culture dish (MatTek) for 24 h. Cell membranes and DNA were stained with CellMask (red) and DAPI (blue), respectively. Confocal reflection microscopy (to visualize HZ phagocytosed by BMDM) combined with fluorescence microscopy was used as described in detail in [26], on a Leica TCS SP2 AOBS confocal laser-scanning microscope.

### Stimulation of bone marrow derived macrophages for nitric oxide induction

BMDM were seeded and incubated overnight (96-well plates, 1 × 10^5^ cells/well). Next day, the cells were primed with 12.5 U/ml Type I Interferon-gamma (IFN-γ) for 2 h, or left untreated, and then stimulated with different concentrations of HZ (12.5, 25, 50, 100 µM). Muramyldipeptide (MDP) (2 µg/ml) or lipopolysaccharide (LPS) (200 ng/ml) were used as positive controls. After 24 h of incubation, the supernatants were collected and used for NO determination by the Griess reagent. These supernatants are referred as “24 h”. Fresh medium was then added and an extra incubation time of 24 h was carried out. The “24 h + 24 h” supernatants were collected and assayed for NO content. In some experiments, fresh medium was added again, further 24 h incubation was performed and the “24 h + 24 h + 24 h” supernatants collected for NO measurement by Griess reagent.

### Experiments with l-arginine-enriched media

BMDM were seeded as explained above. Medium was replaced with fresh standard medium or with medium in which l-Arg was added to obtain ten-fold concentration of l-Arg (840 mg/l) (l-Arg 10×) with respect to the l-Arg content (84 mg/l) of the standard media formulation (DMEM). Respective media (standard medium or l-Arg 10×) were used for all the experimental steps described below. Cells were primed for 2 h with 12.5 U/ml IFN-γ, and then treated with medium, HZ (25 or 100 µM), MDP (2 µg/ml), or LPS (200 ng/ml) for 24 h or 24 h + 24 h. At each time point, the production of NO was determined by the Griess reagent.

### Griess assay for nitric oxide determination

To measure NO production, the indirect measure of the accumulation of nitrite in the cell supernatants was evaluated by the Griess reagent, a standard assay largely used in different models [28, 31–33]. The Griess reagent consists of a mixture of equal parts of Reagent A (1% [w/v] sulphanilamide), and Reagent B (0.1% [w/v] naphthylethylenediamine dihydrochloride, and 2.5% [w/v] phosphoric acid). One hundred microliters of supernatants were mixed with an equal volume of Griess mixture and after 10 min of incubation at room temperature, the nitrite levels were measured by a spectrophotometer (Synergy 4 microplate reader, Biotek^®^, GE) at 540 nm. To determine the levels of nitrite, a standard curve of NaNO_2_ was prepared in parallel to the samples.

### Experiments with haemozoin-conditioned medium

Medium alone or in the presence of HZ (200 µM) was incubated overnight at 37 °C under an atmosphere of 5% CO_2_. HZ-treated medium was centrifuged (3000 rpm, 10 min), and supernatants were filtered (0.20 µm/PSFV). The presence of free haem was excluded spectrophotometrically, as described above. The HZ-treated medium will be referred as HZ-conditioned medium (HZ-CM). The medium alone (untreated conditioned medium, U-CM) received the same handling as the HZ-CM.

BMDM were seeded and incubated overnight, as described above. The medium was then replaced with U-CM or HZ-CM. Then, both priming with IFN-γ and the treatments with HZ (25 or 100 µM), or MDP (1 or 2 µg/ml) were performed in U-CM or HZ-CM. The plates were incubated for 24 h and the levels of nitrite were determined in the supernatants by the Griess reagent.

### Determination of reactive oxygen species release by stimulated bone marrow derived macrophages

BMDM were seeded in 96-well plates (1 × 10^5^ cells/well), at 37 °C under an atmosphere of 5% CO_2_. After 24 h, cells were primed or not with IFN-γ 12.5 U/ml for 2 h, and then stimulated with medium, HZ (25 or 100 μM), MDP (2 μg/ml), or tert-butyl hydrogen peroxide (t-BHP, 10 µM). After 0.5, 18 or 48 h, cells were washed with PBS, and then the levels of reactive oxygen species (ROS) were measured by treating stimulated BMDM for 15 min with 50 µl of 2′7′-dichlorofluorescein diacetate (H_2_DCFDA, 15 µM). Then, the cells were washed with PBS, and 200 µl PBS were added to each well for 30 min; 150 µl of supernatants were transferred into a flat-bottom black plate, and ROS were measured, and recorded as fluorescence units (FU) (Ex/Em:485 nm/535 nm; Synergy 4, Biotek^®^).

### Western blotting

BMDM were plated in 6-well plates (3 × 10^6^ cells/well), at 37 °C under an atmosphere of 5% CO_2_. At the end of an overnight incubation, the cells were primed or not with IFN-λ for 2 h and then treated with medium, HZ (25 or 100 μM), or MDP (5 μg/ml). The plates were incubated for 6 h, 24 h, or 24 h + 24 h. After each time point, cells were washed several times in ice-cold PBS, lysed in RIPA buffer (50 mM TRIS–HCl [pH 7.5], 105 mM NaCl, 1% NP-40, 1% sodium deoxycholate, 0.1% sodium dodecyl sulfate [SDS] and 2 mM ethylenediamine tetraacetic acid [EDTA]) and protease inhibitor cocktail, and incubated on ice for 15 min. The lysate was centrifuged at 15,000 rpm for 15 min at 4 °C. Equal amounts of proteins of each sample supernatant (50 µg), determined by Bradford method [34], were separated by electrophoresis on 7.5% SDS–polyacrylamide gel (SDS-PAGE). Electrophoresed proteins were transferred onto PVDF membranes (Bio-Rad, Richmond, CA, USA) with the Bio-Rad Transfer Blot Apparatus at 150 mA for 16 h at 4 °C using 25 mM Tris HCl, 190 mM glycine, 20% methanol, and 0.05% SDS as transfer buffer. The membranes were then blocked for 2 h and 30 min in blocking buffer (TTBS: 0.5% Tween-Tris-buffered-saline) with 5% bovine serum albumin (SIGMA, St. Louis, MO, USA)]. The blots were incubated with the specific primary antibody (mouse polyclonal anti-iNOS antibody), and mouse monoclonal anti-Actin antibody for 16 h at 4 °C in blocking buffer, washed 6 times for 5 min each in TTBS and incubated with the proper secondary horseradish peroxidase (HRP)-linked IgG antibodies (donkey anti-rabbit and goat anti-mouse), at room temperature in blocking buffer. After 1 h incubation, the membranes were washed and incubated again in TTBS, and finally developed with the ECL western blotting detection reagents (GE Healthcare Life Sciences, UK) following the instructions of the manufacturer. Immunoreactive proteins were visualized by autoradiography on Amersham Hyperfilm™ ECL™ (GE Healthcare Life Sciences, UK).

Quantitative analyses of immune reactive proteins were performed by digital scanning (CAMAG VideoStore 3.00.0.06 serial n° 0607C003 and VideoScan TLC/HPTLC Evaluation Software version 1.01.00 serial n° 0607D003).

### Experiments with liquid chromatography–tandem mass spectrometry

A quantitative analysis of l-Arg in the medium was done through the liquid chromatography–tandem mass spectrometry **(**LC–MS/MS) technique described elsewhere [35]. Briefly, standard medium (MED) or medium seven times more concentrated in l-Arg than standard medium (MED l-Arg 7×) were prepared as controls. A final concentration of 200 μM of HZ was prepared as HZ-CM in standard culture medium or in MED l-Arg 7× (HZ-CM l-Arg 7×). l-Arg (40 ng/ml) was used as control.

The time retention values, converged in the peak areas (area), were evaluated for each sample. Area values were determined for an internal control of l-Arg (40 ng/ml), medium (untreated conditioned medium, U-CM), medium treated with HZ 100 μM (haemozoin-conditioned medium, HZ-CM), medium enriched with l-Arg (U-CM l-Arg 7×), or HZ-CM enriched with l-Arg (HZ-CM l-Arg 7×). Before the chromatographic analyses, samples were prepared and incubated at 37 °C for 24 h under an atmosphere of 5% CO_2_. Next, media were centrifuged and filtered (0.20 µm/PSFV). The area corresponding to the content of l-Arg exhibited by each treatment was retrieved. The mobile phases used are displayed in Table 1.

**Table 1:** LC–MS/MS phases

Time (min)	Phase A	Phase B
0	100	0
5	100	0
8	0	100
20	0	100
21	100	0
28	100	0

The mobile phases of the LC–MS/MS were water plus formic acid 0.1% (Phase A), and CH_3_CN plus formic acid 0.1% (Phase B). Gradients, at a flux 0.3 ml/min, were passed through the chromatographic column (Waters Atlantis C185 µm)

The instrument conditions were as follows: capillary temperature 275 °C; spray voltage 5.00 kV, sheath gas flow rate 60 (arb); auxiliary gas flow rate 15 (arb). Numbers in columns (“Phase A” and “Phase B”) represent the percentage (%) of the respective eluent.

The quantifier and qualifier transition for l-Arg was 175 > 158. Electrospray ionization in positive mode (ESI +) experiments was performed using a tridimensional ion trap-mass spectrometer (DECA XP MAX, Thermo Fisher). The data were acquired by means of XCalibur (Thermo Fisher) software.

### Statistical analyses

Differences between groups were analyzed by one-way or two-way ANOVA analysis and post-hoc multiple comparisons tests (Bonferroni, Sidak, Tukey), using the software GraphPad Prism 6. Data are representative of at least three independent experiments in triplicate.

## Results

### Nitric oxide production by interferon gamma primed macrophages stimulated with haemozoin follows a bell-shaped, dose–response curve

**Fig. 1:**
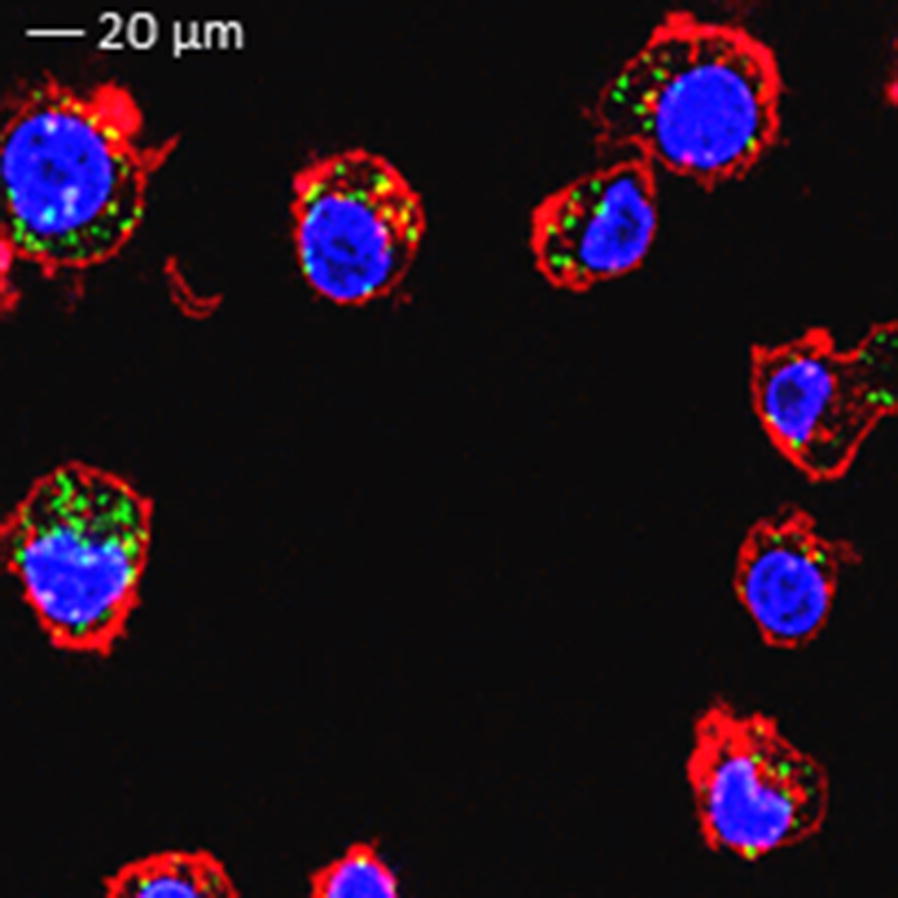
Phagocytosis of HZ by bone marrow derived macrophages. BMDMs were incubated with HZ (100 µM) for 24 h. Cell membranes and DNA were stained with cell mask (red) and DAPI (blue), respectively. Confocal reflection microscopy was used to visualize HZ (green). Scale bar: 20 μm. The Leica TCS SP2 AOBS confocal laser-scanning microscope was set to allow 10% of laser light into the collection channel. Fields are representative of at least ten fields of view and three independent experiments

**Fig. 2:**
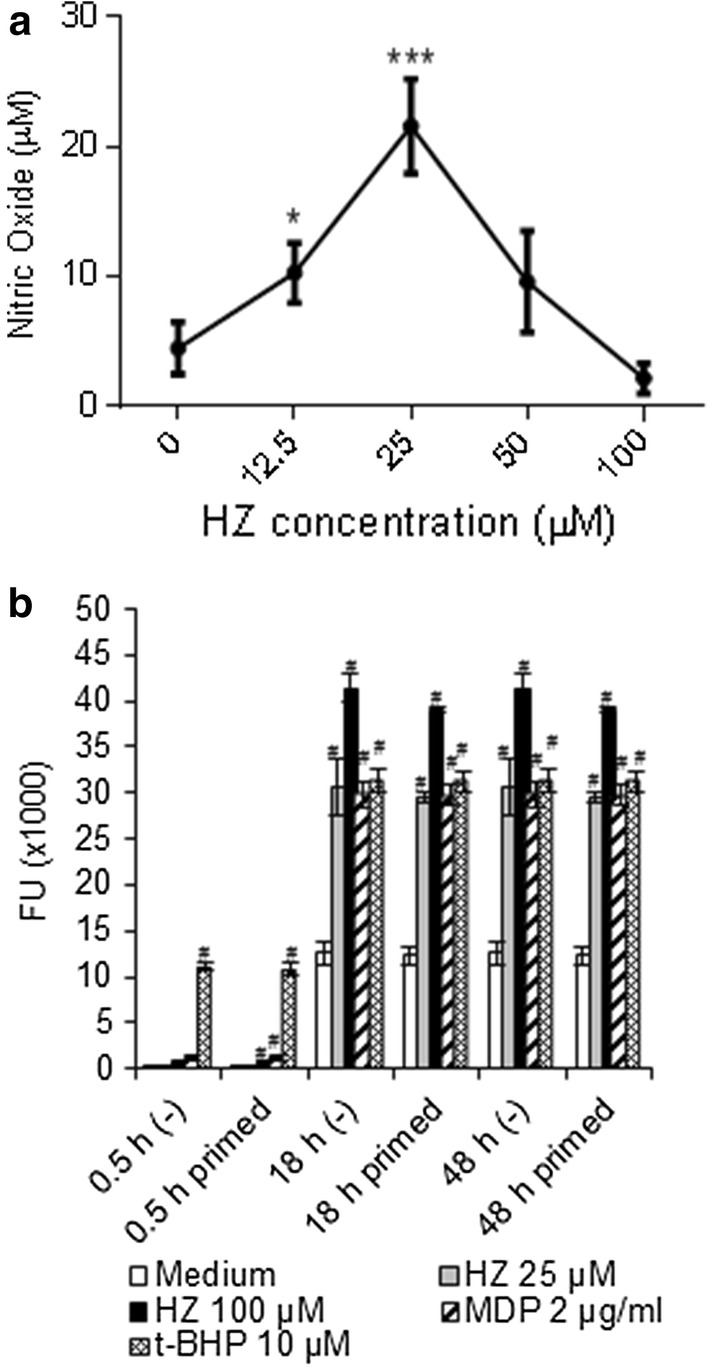
Levels of NO and ROS induced by HZ in BMDM. BMDM were primed with IFN-γ for 2 h, and then treated with medium or different concentrations of HZ (12.5, 25, 50, 100 µM). Levels of nitrite (vertical axis) released into the supernatants after 24 h were measured. ^*^P < 0.01, ^*** #^*P* < 0.0001 vs control medium. Data displayed as averages ± SD of three independent experiments (**a**). BMDM were primed or not for 2 h. Cells were then stimulated with HZ (25 or 100 µM), MDP (2 µg/ml), or tert-butyl hydroperoxide (t-BHP) (10 µM). After each time point (0.5, 24 or 48 h) the supernatants were discarded and the cells were treated with 2′,7′-dichlorofluorescein diacetate (DCFDA) (15 µM) for 30 min at 37 °C. The cellular oxidative stress induced in macrophages was measured using the Synergy4 (BioTek) fluorescent microreader and the fluorescent units (FU, vertical axis) were recorded and correlated to the levels of ROS produced. ^#^*P* < 0.0001 vs control medium. Data represent averages ± SD of three independent experiments (**b**)

BMDM were treated for 24 h with HZ, which was readily phagocytosed, as evaluated by confocal microscopy (Fig. 1). Priming of BMDM with IFN-γ, shown to be required for the production of nitric oxide (NO) upon HZ stimulation, was always performed before treatment with different concentrations of HZ [28]. Detectable levels of NO were observed at 24 or 48 h but not at 6 h of incubation, therefore the majority of the experiments were done at 24 h. The nitrite levels released into the supernatants were evaluated as a measure of NO production. Figure 2a shows that doses of HZ up to 25 µM linearly increased NO production, but, unexpectedly, higher doses, from 50 to 100 µM, induced an inverse dose–response.

To exclude that the decrease in nitrite levels was due to a possible oxidation of nitrite to nitrate by HZ, in some experiments, all nitrate were converted into nitrite and the total nitrite levels in the supernatants were measured using the Griess reagent. The results were comparable to those obtained measuring only nitrite (Additional files 1, 4), indicating that the lower amount of nitrite observed with high doses of HZ was not due to the oxidation of nitrite to nitrate.

It has been shown that ROS can modulate NO production [36], and that both HZ [37] and βH, the synthetic HZ, induce ROS [38]. Thus, to investigate whether oxidative stress could be involved in the lower levels of NO found in the supernatants of BMDM treated with increasing concentrations of HZ, ROS release by primed or unprimed BMDM was tested. The lowest (25 µM) and the highest dose (100 µM) of HZ were chosen from the previous experiments. In addition, cells treated with medium only or activated by control agents such as MDP, a bacterial cell walls component [39], or tert-butyl hydrogen peroxide (t-BHP) were included. Tert-butyl hydrogen peroxide was used as a positive control since it is an organic peroxide, able to induce ROS formation to levels similar to hydrogen peroxide, but with less cellular toxicity [40]. As shown in Fig. 2b, at 0.5 h HZ induced low but significant levels of ROS only at 100 µM, but not at 25 µM. At 18 h, HZ induced a dose-dependent increase of ROS. This activity was independent of priming, as there were no differences in unprimed or primed cells. In addition, MDP or t-BHP stimulated the production of ROS in the same manner at 18 h (Fig. 2b). After 48 h incubation, the levels of ROS induced by all stimuli did not change significantly compared to 18 h, indicating that ROS produced from activated BMDM reached a plateau concentration already at 18 h. Collectively, these data indicated that, at all time points, HZ induced ROS in a dose-dependent manner. This result does not reflect the inverse dose–response of NO production induced by HZ, reported in Fig. 2a.

It has been described that free haem can compromise the transport of l-Arg in the erythrocytes [23]. To exclude a possible involvement of free haem in the regulation of NO production, HZ was extensively washed and the presence of free haem, as well as of haemoglobin, was measured and excluded in any preparations used.

Since l-Arg is a substrate also for Arginase-1, the activation of this enzyme by HZ was also excluded. The levels of urea, the main product of arginase activity, in cellular extracts from macrophages treated with HZ were comparable to those observed in untreated cells (Additional files 2, 4). In addition, in murine macrophages, arginase-1 is known to be regulated by Th2 cytokines, such as IL-4, IL-10, and IL-13 [41–43], whereas IFN-γ is needed for iNOS up-regulation. In our mouse macrophages model IFN-γ was used for iNOS induction and thus for NO release from treated macrophages.

### Expression of inducible nitric oxide synthase does not match nitric oxide production by macrophages treated with different concentrations of haemozoin

**Fig. 3:**
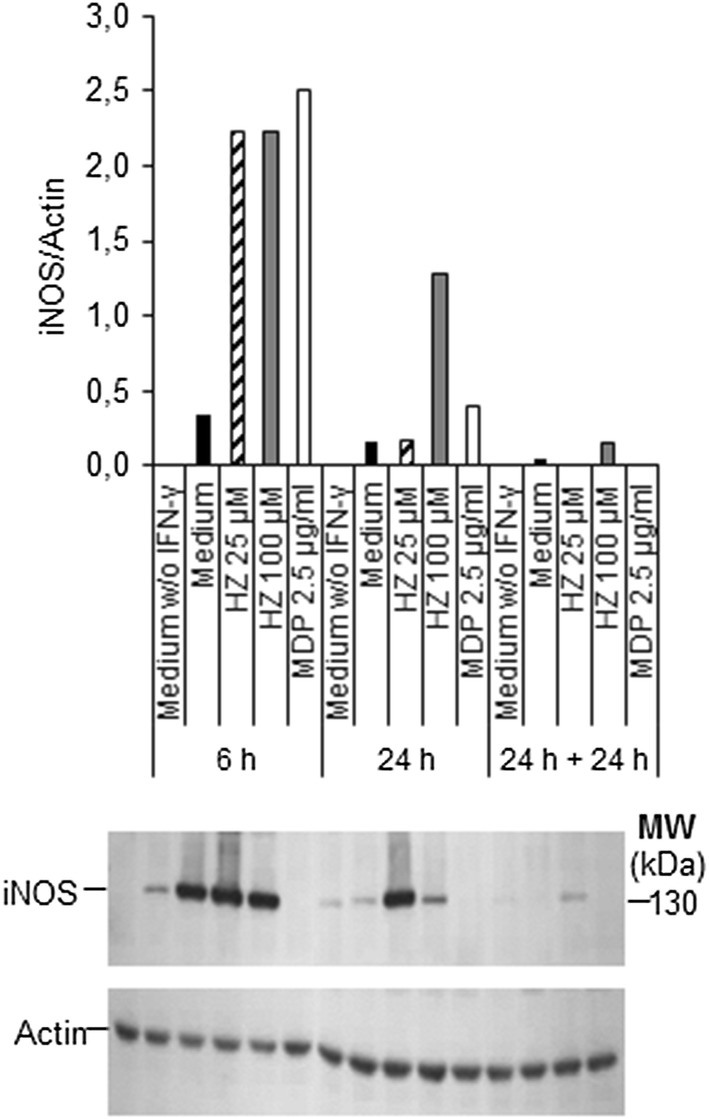
Levels of iNOS protein and of NO detected in HZ-treated BMDM. BMDM were primed or not for 2 h. Cells were treated with medium, HZ (25 or 100 µM), or MDP (2.5 µg/ml). Cell lysates were collected after 6, 24, or 24 h + 24 h. Equal amounts of proteins for each sample were electrophoresed, and transferred onto PVDF membranes. iNOS and actin proteins were probed with their respective antibodies. Vertical axis shows the ratios of iNOS/actin values. Results shown are representative of three independent experiments

To investigate whether HZ, according to the concentration used, induces iNOS expression in BMDM, the levels of iNOS were assessed at different time points by western blotting. As expected, unprimed macrophages did not express iNOS at any time point tested, while primed cells expressed low levels of iNOS even in the absence of a stimulus (Fig. 3). Primed macrophages treated for 6 h with HZ at 25 or 100 µM expressed high iNOS levels, comparable to those induced by MDP. The levels of iNOS peaked at 6 h, with a progressive decrease at the additional time points. At 24 h and at 24 h + 24 h, after washing and medium replacement, HZ induced iNOS in BMDM only at the dose of 100 μM. Levels of iNOS expression in cells treated with MDP were higher than medium at both 6 and 24 h, but disappeared at 24 h + 24 h. Overall, the iNOS expression in BMDM stimulated with HZ followed a kinetic similar to that induced by MDP (i.e. decrease in iNOS expression over time) and was dose-dependent. This is at variance with what observed for the NO levels and indicates that the production of NO by BMDM stimulated with HZ does not reflect the levels of iNOS.

### Fresh medium replacement restores nitric oxide production by macrophages treated with haemozoin in a dose–response manner

**Fig. 4:**
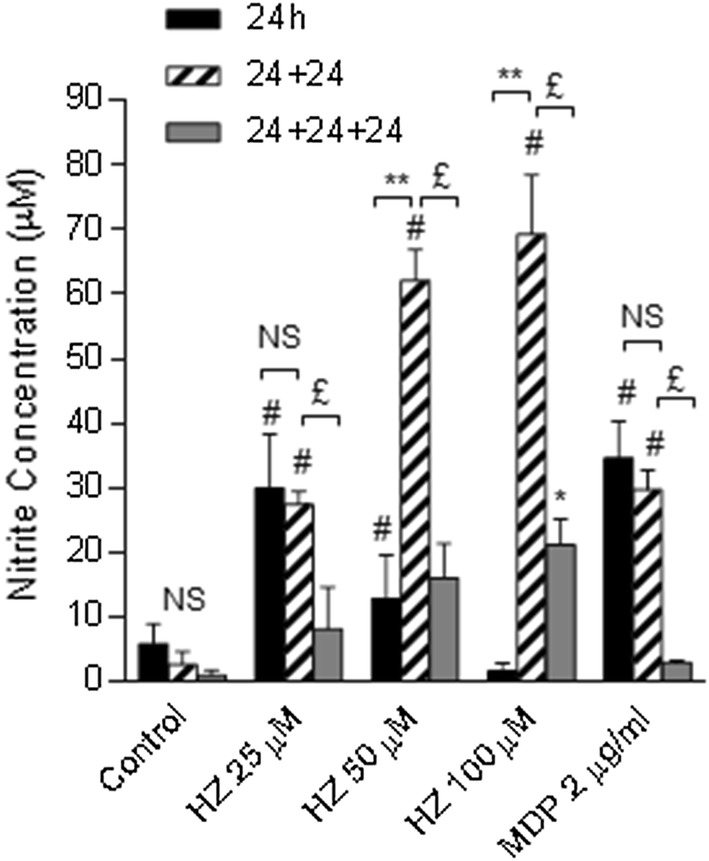
NO production by BMDM stimulated with HZ after medium replacement. BMDM were primed for 2 h, and treated with HZ (25, 50, or 100 µM), or MDP (2 µg/ml). After 24 h incubation, the supernatants were removed and the levels of nitrite were determined. Fresh medium was added to the cells and further 24 h incubation was performed (24 h + 24 h). Supernatants were collected again, and the nitrite levels determined. Incubation with fresh medium was carried out for further 24 h (24 h + 24 h + 24 h). The supernatants were collected at the end of incubation, and the nitrite levels were determined. ^#^*P *< 0.01 and ^*^P < 0.05 vs control medium at each time point; ^**^P < 0.01 24 h + 24 h vs 24 h; ^£^*P* < 0.01 24 h + 24 h vs 24 h + 24 h + 24 h. *NS* not significant. Data represent averages ± SD of three independent experiments

To investigate the inverse relationship between the doses of HZ and NO production, primed BMDM were treated with medium, different concentrations of HZ, or MDP for 24 h. At the end of the incubation, the supernatants were collected and used for NO determination. Fresh medium was then added to both stimulated and unstimulated cells. One or two extra incubation times of 24 h each where then carried out, the supernatants recovered and assayed for NO content. As depicted in Fig. 4, the levels of NO released during the first 24 h were inversely related to the HZ dose, whereas NO induced by MDP was significantly higher than medium. Surprisingly, and opposite to what observed after the first 24 h, the levels of NO detected in the 24 h + 24 h supernatants increased considerably compared to the first 24 h and were proportional to the dose of HZ used. Moreover, in the supernatants recovered at the end of the following 24 h of incubation (i.e. 24 h + 24 h + 24 h), the levels of NO were lower than at 24 h + 24 h, but retained a direct proportionality with the dose of HZ. The release of NO by cells treated with MDP at both time points was comparable to that at 24 h. It seems thus that medium replacement after the first 24 h of stimulation of HZ restores the dose–response for NO production, which seems to peak after 24 h + 24 h and decreases in the following 24 h.

### Excess of l-arginine in the media restores nitric oxide production by haemozoin

**Fig. 5:**
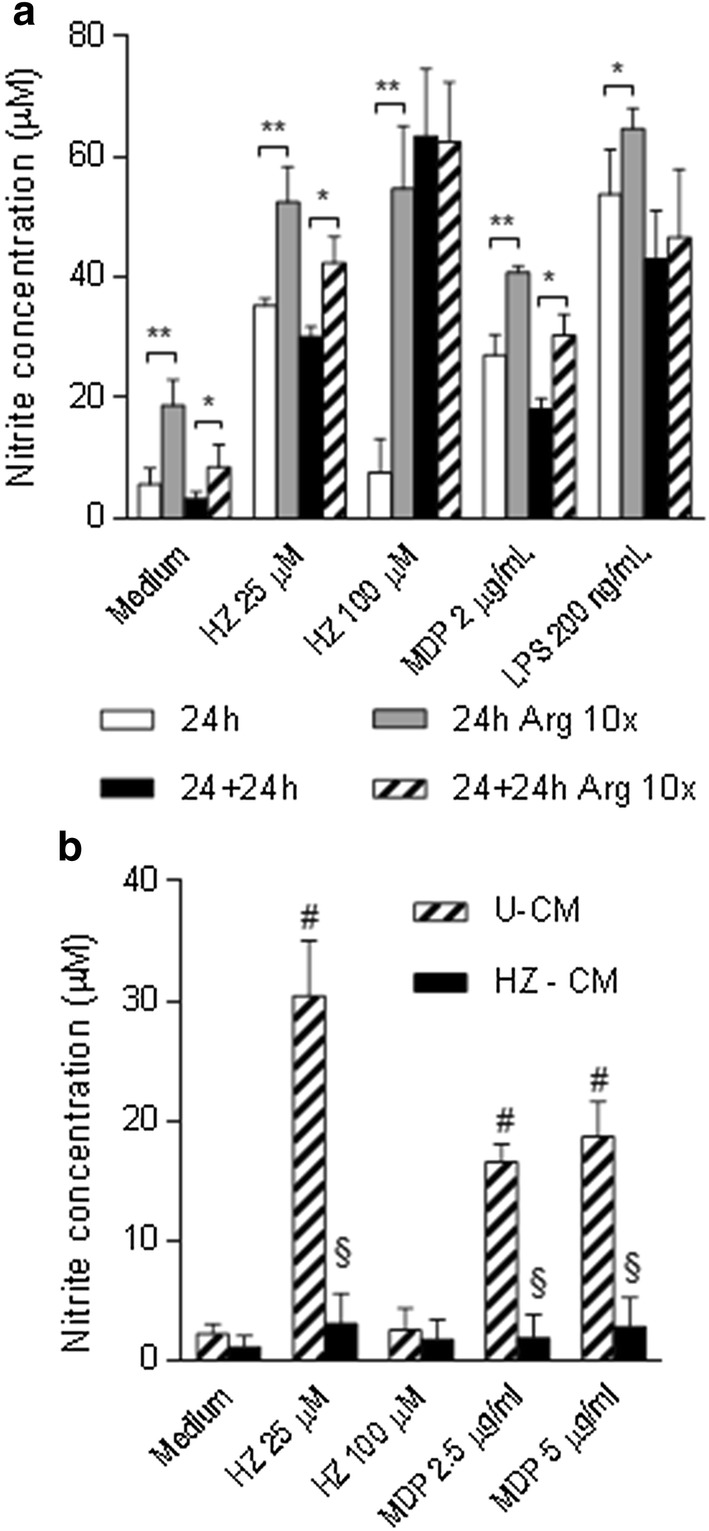
NO production by BMDM treated with HZ and cultured in medium enriched in l-Arg (**a**) or in HZ-conditioned medium (**b**). BMDM were plated overnight in either standard medium or medium enriched with l-Arg (l-Arg 10×). Respective media (standard medium or l-Arg 10×) were used for all the experimental steps described below. Cells were primed for 2 h, and then stimulated with medium, HZ (25 or 100 µM), MDP (2 µg/ml), or LPS (200 ng/ml) for 24 h. Levels of nitrite were determined, and fresh medium was replaced. After additional 24 h incubation (24 h + 24 h), the nitrite levels in the supernatants were determined again. ^*^P < 0.05 and ^**^P < 0.01 vs standard medium at each time point. Data displayed as averages ± SD of three independent experiments (**a**). BMDM were plated overnight in either standard medium (unconditioned medium, U-CM) or in HZ-conditioned medium (HZ-CM). Respective media (U-CM or HZ-CM) were used for all the experimental steps described below. Cells were primed for 2 h, and then stimulated with medium, HZ (25 or 100 µM), or MDP (1 or 2 µg/ml) for 24 h. Levels of nitrite were measured in supernatants. ^#^*P* < 0.01 vs control medium, ^§^*P* < 0.01 vs U-CM. Data displayed as averages ± SD of three independent experiments (**b**)

The observation that media replacement after 24 h restored the dose-dependent production of NO by HZ-treated BMDM and that iNOS was not a limiting factor led to the hypothesis that the availability of l-Arg, the substrate of iNOS, could be low or reduced in the groups where HZ was also present. To verify this hypothesis, the experiments were repeated by stimulating BMDM using an l-Arg-enriched medium in which l-Arg was added to reach final concentration ten-fold higher than that of the standard medium (l-Arg 10×). The levels of NO were evaluated both after 24 h and after medium change at 24 h + 24 h (Fig. 5a). In all cases, the stimulation of primed BMDM with medium, HZ (25 or 100 µM), MDP, or LPS led to an increase of NO production. Using medium with l-Arg 10×, a further increase of NO production was seen in all groups, and, in particular, in the sample treated with 100 µM of HZ, which did not induce NO in the standard medium. The dose of HZ was positively correlated with the production of NO, and opposite to what observed in normal medium conditions. Therefore, it appears that an excess of l-Arg in the medium restores the production of NO induced by high HZ doses (100 µM), indicating a possible interaction between l-Arg and HZ.

### Haemozoin decreases nitric oxide production by limiting l-arginine availability

To investigate the ability of HZ to directly reduce l-Arg availability, a HZ treated-conditioned medium (HZ-CM) was prepared, as described in the “Methods” section. Untreated conditioned medium (U-CM) was also prepared. BMDM were treated with different concentrations of HZ or MDP either in U-CM or HZ-CM. The production of NO by both doses of HZ or MDP was abolished when the stimuli were prepared in HZ-CM, but not in U-CM (Fig. 5b). Being l-Arg the substrate of iNOS, these results suggested that HZ might interact with l-Arg, present in the culture medium, depleting the medium of the substrate for iNOS, thus reducing NO production.

To measure the effective presence of l-Arg, U-CM and HZ-CM were analysed by LC–MS/MS. A control of l-Arg (40 ng/ml) was run on the column (Table 2), exhibiting a peak area of 384,603. The peak area of U-CM, which contains 40 ng/ml of l-Arg, was 501,886. In contrast, in the HZ-CM no l-Arg was detected (Table 2). To better quantify the amount of l-Arg that interacted with HZ, a preparation of HZ-CM and U-CM was made using a medium containing l-Arg 7 times more concentrated than in normal medium (HZ-CM l-Arg 7× and U-CM l-Arg 7×, respectively). The samples were then run in a LC–MS/MS column and it was found that the peak area exhibited by U-CM l-Arg 7× was 1.7 times higher than HZ-CM l-Arg 7×.

**Table 2:** Determination of l-Arg in different samples of medium through LC–MS/MS

Sample^a^	Area^b^
l-Arg^c^	384,603
U-CM	501,886
HZ-CM	0
U-CM l-Arg 7×	3,418,162
HZ-CM l-Arg 7×	1,988,197

^a^Samples injected to the columns of the LC–MS/MS

^b^Area (expressed as arbitrary unit) calculated from the LC–MS/MS

^c^l-Arg at a concentration of 40 ng/ml

## Discussion

The present study provides the first in vitro evidence of the interaction between HZ and l-Arg, leading to lower availability of l-Arg, the substrate for iNOS, and thus reduced production of NO by activated macrophages.

This finding came from an unexpected observation that the levels of NO released by activated BMDM followed a bell-shaped curve, as the HZ concentration increased. At doses of HZ above 25 µM the production of NO progressively declined. This phenomenon was not related to ROS production, or to decreased iNOS expression in macrophages. The NO scavenger effect of haem or haemoglobin was excluded since all the HZ preparations were extensively washed and resulted free of haem or haemoglobin contamination. The linear dose–response of NO production by HZ was restored by changing medium or adding 10× excess of l-Arg in the culture, suggesting a depletion of l-Arg from the culture medium more than a problem of macrophage activation. Subsequent experiments by LS–MS–MS indeed demonstrated that purified HZ deprives the medium of l-Arg, probably through a molecular interaction between HZ and l-Arg.

HZ is an insoluble crystal consisting of Fe(III)PPIX (haem) bound to proteins, lipids or DNA of parasite or host origin [44–46]. Once released at schizont rupture, it is rapidly taken up by phagocytic cells (see Fig. 1), without being degraded [47]. It has been demonstrated that HZ can affect the function of macrophages through different pathways such as TLR9 or NOD2 pathway [28, 44, 48], or NLRP3 [25, 49]. Both stimulatory and inhibitory immune properties of HZ in vitro have been described and depend on the source of macrophages, the protocols and the co-stimuli used for the functionality assays, and the methods of isolation and purification of HZ from parasite cultures [25, 32, 38, 44]. In the present study, to better mimic the in vivo situation, only the pigment released free at schizont rupture into the *P. falciparum* culture medium was selected, purified and used in the assays. Nonetheless, additional variables, namely the constituents of the culture media or supplements (like serum), may account for the differences observed in functionality experiments.

Among its immunomodulating activities, HZ can induce the production of NO by mouse or human macrophages from different sources [28, 38, 50]. In macrophages, both eNOS and iNOS isoforms are present and can produce NO after homodimerization with other molecules such as haem, calmodulin, tetrahydrobiopterin, flavins [51]. The levels of iNOS in macrophages are significantly higher than those of eNOS [52], and therefore more relevant in the present model. Here, it is shown that iNOS expression was not congruent with NO levels released from macrophages stimulated with different concentrations of HZ. This observation is in accordance with clinical data from a study performed in Papuan (Indonesia) patients showing no correlation between iNOS activity in peripheral blood mononuclear cells and plasma concentrations of nitrite and nitrate [53].

Phagocytosed HZ can destabilize the phagosome integrity, as hypothesized by Kalantari and colleagues [25]. Thus, the presence of HZ in the cytosol may deprive the cells from intracellular l-Arg. Chemically, the interaction between HZ and l-Arg is not unlikely. The HZ crystal is highly ordered with a known structure [29]. The end faces of the needle-like crystals expose haem propionate groups. The hydrogen bonding interaction between the carboxylate group of a propionate and the guanidinium side chain of arginine is very strong because of the presence of two parallel H-bonds with the added electrostatic interaction arising from the positive charge of the guandinium group attracting the negative charge of the carboxylate group. Thus, an interaction between HZ and l-Arg can be expected. Indeed, Uyen and colleagues showed that basic amino acids, such as l-Arg or l-Lysine (l-Lys), inhibit the formation of βH [24], the synthetic crystal made only of the haem portion of HZ, indirectly demonstrating the interaction of l-Arg with haem. However, in the present experiments, synthetic βH, differently from native HZ, was not able to reduce NO production even at the dose of 60 µM (Additional files 3, 4) suggesting that the l-Arg–HZ interaction requires also a component of HZ, not yet identified. In any case, it is difficult to imagine that this depletion can arise from anything else than binding of l-Arg to HZ (or molecules associated with HZ), since no other component differs between the control and HZ containing media. Direct methods to visualize l-Arg binding to HZ are not available since HZ is a macroscopic crystal approximately 0.5 µM in length and l-Arg is a small molecule. Adsorption of l-Arg from the medium is the only practical way to demonstrate such interaction. The fact that the addition of excess l-Arg to the culture medium results in incomplete depletion, suggests a saturated binding.

The observation that HZ removes l-Arg from the culture medium might have clinical implications, as high release of HZ, low levels of l-Arg and/or NO are reported to be associated with severe malaria [7, 8, 11, 19–22, 54–56].

How the bioavailability of NO contributes to the status of severe malaria is still controversial [11, 13]. Disease severity in Tanzanian children has been associated with low levels of NO [54]. This might result in microvascular obstruction, tissue ischemia, and clinical complications. Further evidence is provided by animal models: protection against experimental cerebral malaria has been reported by treatments with inhaled NO or NO donors [14, 15].

Host components have been linked to low levels of NO during malaria: free haemoglobin, from burst of infected erythrocytes or intravascular hemolysis, which scavenges NO [57]; the activation of arginase, in infected or uninfected erythrocytes, which catalyzes the production of ornithine and urea from l-Arg [58]; the presence of endogenous iNOS inhibitors such as methylated arginines (asymmetric dimethylarginine [ADMA] and symmetric dimethylarginine [SDMA] [59]); the decreased levels of tetrahydrobiopterin, which uncouples iNOS [60]. Yet, the availability of l-Arg, the iNOS substrate, would definitely represent the limiting factor for NO production.

Hypoargininaemia is a common feature exhibited in both children and adults with severe malaria [19, 20, 61]. One of the proposed causes, although controversial, of l-Arg depletion is the increase of arginase activity generated by parasites or host cells [58, 62, 63], but no report in the literature seems to link malaria haemozoin and arginase-1 activation, so far. In addition, the results here showed that HZ does not seem to activate this enzyme. Hypoargininaemia may also derive from an excess of free haem which dysregulates the metabolism and the availability of l-Arg. An earlier report from our group shows that free haem increases intra- and extra-erythrocyte l-Arg [23]. The hypothesis proposed in this study is that HZ release into the circulation can contribute to hypoargininaemia. This is plausible, since low levels of l-Arg have been associated to high parasitaemia and severe malaria, and both conditions are associated to high levels of HZ. It is conceded here that HZ may not constitute the exclusive cause of hypoargininaemia or of decreased levels of NO in malaria, as a number of other factors could contribute to this activity, too. Hitherto, the data presented here might bring new insights into the pathogenesis of the disease concerning the relationships between severe cases of malaria and HZ accumulation in leukocytes or bone marrow [64]. T-lymphocyte functions are also regulated by l-Arg bioavailability [65] thus hypoargininaemia might also affect adaptive immunity during malaria.

The use of l-Arg for adjunctive therapy during cerebral malaria has been questioned; however, several reports show its relevance as a complement for antimalarial therapy during the early course of the infection [11], as enhancer of NO production, well known for its antiadhesive properties [5]. Hypoargininaemia during malaria infections has been also correlated with reduced *P. falciparum* erythrocytes deformability [10]. This may be relevant for the gametocytes, the transmission stage of malaria parasites, which sequester and develop in the bone marrow, from where they egress [66, 67] as a consequence of increased deformability [68, 69]. HZ accumulates in the bone marrow [22, 70] where it may interact with l-Arg. Therefore, it is reasonable to propose that HZ, depriving iNOS of l-Arg and thus lowering the production of NO, may also be indirectly involved in the regulation of malaria transmission.

## Conclusions

**Fig. 6:**
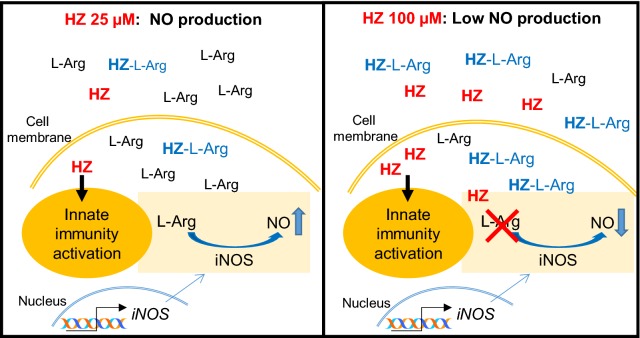
Diagram showing the hypothesis of interaction between HZ and l-Arg. Free or macrophage-phagocytosed HZ may interact with l-Arg (HZ-l-Arg). Depending on the concentration of HZ, different amounts of l-Arg may be trapped, affecting NO production. At 25 µM, HZ does not remove all the l-Arg (iNOS substrate) and NO is produced by macrophages; at 100 µM, HZ removes almost all l-Arg impairing NO production

In conclusion, HZ, at doses higher than 25 µM, reduced l-Arg availability in vitro and thus decreased NO production by IFN-γ-primed BMDM (Fig. 6). These findings could open new insights into the possible causes of hypoargininaemia, and the consequent low NO levels, observed in vivo. However, further investigations are needed to verify the possible implication in clinical severe malaria.

## Additional files

**Additional file 1.** Measurement of nitrite and nitrate concentrations in BMDM treated with malarial hemozoin.

**Additional file 2.** Measurement of urea levels in BMDM treated with malarial hemozoin.

**Additional file 3.** Measurement of nitrite concentrations in BMDM treated with synthetic hemozoin.

**Additional file 4.** Bibliography for Additional files 1, 2, and 3.

## Abbreviations

HZ: natural haemozoin
βH: beta-hematin
BMDM: immortalized bone marrow macrophages
NO: nitric oxide
IFN-γ: interferon-gamma
L-Arg: l-arginine
MDP: muramyldipeptide
LPS: lipopolysaccharide
ROS: reactive oxygen species
TBHP: tert-butyl hydroperoxide
iNOS: inducible nitric oxide synthase
CM: conditioned medium
U-CM: untreated conditioned medium
HZ-CM: haemozoin-treated conditioned medium
LC–MS/MS: liquid chromatography-tandem mass spectrometry
