# Supplementary material for: Interplay between Plasmodium falciparum haemozoin and l-arginine: implication for nitric oxide production
Source: Malar J. 2018 Dec 6;17:456. doi: 10.1186/s12936-018-2602-0 (PMC6282336; doi:10.1186/s12936-018-2602-0)
Supplement: Supplementary file 1 — Additional file 1. Measurement of nitrite and nitrate concentrations in BMDM treated with malarial hemozoin. [file 12936_2018_2602_MOESM1_ESM.pdf]

Additional file 1_Figure

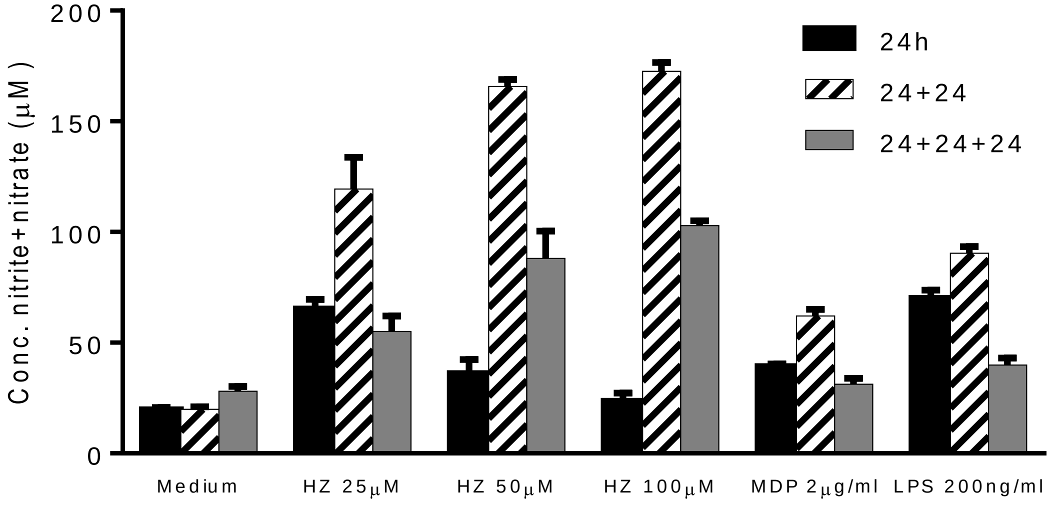

Additional file 1_Legend

Measurement of nitrite and nitrate concentrations in BMDM treated with malarial hemozoin.

BMDM were seeded (96-well plates; 1x10^5 cells/well, in 100 µl of experiment medium) at 37°C under an atmosphere of 5% CO_2, overnight. Cells were then primed with 12.5 U/ml IFN-γ for 2h, stimulated with different concentrations of HZ (25, 50, 100 µM), and MDP (2 µg/ml) or LPS (200 ng/ml) (as positive controls), and incubated for 24h when the supernatants were removed. Fresh medium was added to the cells and a further 24h incubation was performed (24h+24h). Supernatants were collected and an incubation with fresh medium was carried out for further 24h (24h+24h+24h), when the supernatants were collected. In the supernatants collected at any time point, concentrations of both nitrite and nitrate were measured, converting nitrate to nitrite as described in the protocol of Miranda and colleagues [1]. Briefly, 100 µl supernatants, 100 µl of vanadium chloride (VCl3), and 100 µl of the Griess reagent were mixed, and incubated for 30 minutes at 37°C. The levels of nitrite and nitrate were retrieved through a standard curve of KNO_3 placed in parallel, reading the colorimetric reaction at 540 nm with microplate reader (Synergy 4 microplate reader, Biotek, GE).
