# Supplementary material for: Interplay between Plasmodium falciparum haemozoin and l-arginine: implication for nitric oxide production
Source: Malar J. 2018 Dec 6;17:456. doi: 10.1186/s12936-018-2602-0 (PMC6282336; doi:10.1186/s12936-018-2602-0)
Supplement: Supplementary file 2 — Additional file 2. Measurement of urea levels in BMDM treated with malarial hemozoin. [file 12936_2018_2602_MOESM2_ESM.pdf]

Additional file 2_Figure

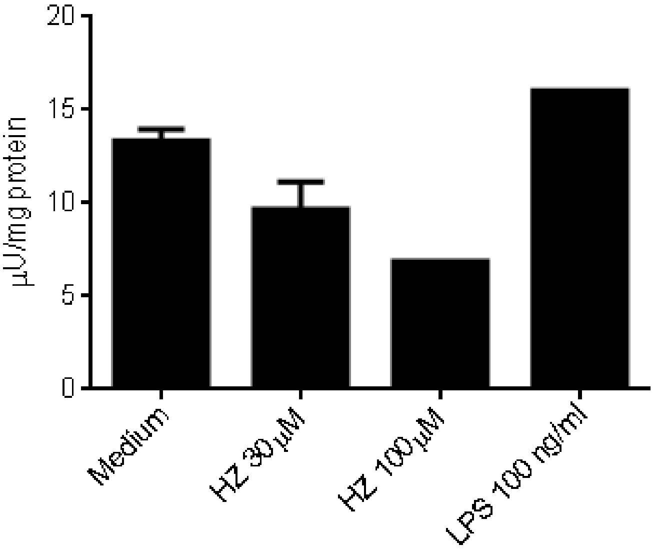

Additional file 2_Legend

Measurement of urea levels in BMDM treated with malarial hemozoin.

BMDM were seeded (24-well plates; $2.5x10^5$ cells/well, in 500 µl of experiment medium) at 37°C under an atmosphere of 5% CO_2, overnight. Cells were primed with 12.5 U/ml IFN-γ for 2h, and then stimulated with different concentrations of HZ (30, 100 µM), or LPS (200 ng/ml). After 24h incubation, the cells were washed with PBS and lysed in a cell lysis buffer (Cell Signaling Technology®). The concentration of protein of each sample was determined through Bradford reagent (Biorad®) [2].

The cell lysate was incubated with 0,25 M L-Arg (pH 9,7) for 30 min at 37°C.

The reaction was stopped by adding an acid mixture (H_2SO_4-H_3PO_4-H_2O, 1:3:7, vol/vol/vol). Arginase activity in the samples was reflected as the quantity of urea formed, which was expressed as the µmol of urea produced per min (µU) per milligram of protein. The urea levels were measured at 540 nm in a spectrophotometer (Synergy 4 microplate reader, Biotek, GE) [3]. Data shown as averages ± SD of three independent experiments.
