# Supplementary material for: Interplay between Plasmodium falciparum haemozoin and l-arginine: implication for nitric oxide production
Source: Malar J. 2018 Dec 6;17:456. doi: 10.1186/s12936-018-2602-0 (PMC6282336; doi:10.1186/s12936-018-2602-0)
Supplement: Supplementary file 3 — Additional file 3. Measurement of nitrite concentrations in BMDM treated with synthetic hemozoin. [file 12936_2018_2602_MOESM3_ESM.pdf]

Additional file 3_Figure

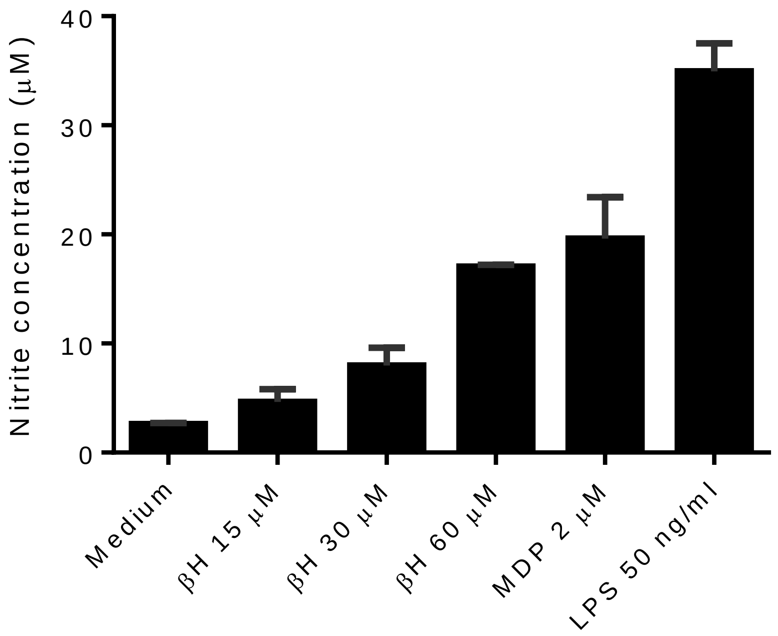

Additional file 3_Legend

Measurement of nitrite concentrations in BMDM treated with synthetic hemozoin.

Beta hemanin (βH, synthetic hemozoin) was prepared as described earlier [4]. BMDM were seeded (96-well plates; $1x10^5$ cells/well, in 100 µl of experiment medium) and incubated at 37°C under an atmosphere of 5% CO_2, overnight. Cells were primed for 2h with 12.5 U/ml IFN-γ, stimulated with different concentrations of synthetic hemozoin (βH) (15, 30, 60 µM), and MDP (2 µg/ml) or LPS (50 ng/ml) as positive controls. After 24h, the levels of nitrite released into the supernatants were measured through Griess reaction. Results are reported as a mean of triplicate determinations ± S.D. (error bars).
