# Supplementary material for: Interplay between Plasmodium falciparum haemozoin and l-arginine: implication for nitric oxide production
Source: Malar J. 2018 Dec 6;17:456. doi: 10.1186/s12936-018-2602-0 (PMC6282336; doi:10.1186/s12936-018-2602-0)
Supplement: Supplementary file 4 — Additional file 4. Bibliography for Additional files 1, 2, and 3. [file 12936_2018_2602_MOESM4_ESM.pdf]

Bibliography for additional files 1, 2, and 3

1. Miranda KM, Espey MG, Wink DA. A rapid, simple spectrophotometric method for simultaneous detection of nitrate and nitrite. *Nitric Oxide*. 2001;5(1):62-71.
2. Bradford MM. A rapid and sensitive method for the quantitation of microgram quantities of protein utilizing the principle of protein-dye binding. *Anal Biochem*. 1976;72:248-254.
3. Corraliza IM, Campo ML, Soler G, Modolell M. Determination of arginase activity in macrophages: a micromethod. *J Immunol Methods*. 1994;174(1-2):231-235.
4. Egan TJ, Chen JY, de Villiers KA, et al. Haemozoin (beta-haematin) biomineralization occurs by self-assembly near the lipid/water interface. *FEBS Lett*. 2006;580(21):5105-5110.
